# Shockwave Therapy or Acupoint Stimulation Combined With Physiotherapy for Spasticity and Motor Function in Post‐Stroke Recovery: A Randomized Controlled Trial

**DOI:** 10.1002/brb3.71652

**Published:** 2026-08-15

**Authors:** Kazi Md Azman Hossain, Meer Hossain Hero, Mahomuda Afroze, Saddam Hossain, Md. Adnan Ibne Reza, Tofajjal Hossain, Ahamadullah Hil Galeb, Jannatul Ferdous Rikti, Mahdi Ul Bari, Arnob Datta

**Affiliations:** ^1^ Department of Physiotherapy and Rehabilitation Jashore University of Science and Technology (JUST) Jashore Bangladesh; ^2^ Department of Physiotherapy Centre For the Rehabilitation of the Paralysed (CRP) Dhaka Bangladesh; ^3^ Japan Bangladesh Friendship College of Physiotherapy and Health Sciences Dhaka Bangladesh

**Keywords:** acupoint stimulation, physiotherapy, shockwave therapy, stroke

## Abstract

**Background:**

Stroke frequently causes spasticity and motor impairment, reducing independence. Non‐invasive treatments like radial extracorporeal shockwave therapy (ESWT) and acupoint‐based transcutaneous electrical nerve stimulation (Acu‐TENS) show potential, but direct comparisons with physiotherapy are limited.

**Aims:**

To investigate the effectiveness of radial ESWT and Acu‐TENS, combined with usual physiotherapy treatment (UPT), in spasticity and motor function during post‐stroke recovery.

**Methods:**

This trial included 120 participants, randomly allocated to three groups (*n* = 40 each): ESWT+UPT, Acu‐TENS+UPT, or UPT alone. Interventions were administered three times weekly for 6 weeks. Primary outcomes included spasticity (Modified Ashworth Scale, MAS), spasticity control (Modified Tardieu Scale, MTS), and motor function (Fugl‐Meyer Assessment, FMA). Secondary outcomes were stroke‐specific quality of life (SS‐QOL) and functional mobility (10‐Meter Walk Test, 10MWT). Assessments were conducted at baseline, post‐intervention (6 weeks), and follow‐up (18 weeks).

**Results:**

Significant time and group effects were observed across all outcomes (p < 0.001). For MAS, ESWT and Acu‐TENS showed non‐significant differences in U/L (6 weeks: MD = −0.175, p = 0.620; 18 weeks: MD = −0.125, p = 1.000) and L/L (6 weeks: MD = −0.075, p = 1.000; 18 weeks: MD = −0.025, p = 1.000). MTS was significantly superior with ESWT in U/L at 6 weeks (MD = 1.584, p = 0.010) and 18 weeks (MD = 1.419, p = 0.022), whereas L/L differences were slightly greater but non‐significant (6 weeks: MD = 1.500, p = 0.122; 18 weeks: MD = 1.500, p = 0.080). FMA was comparable at 6 weeks (U/L: MD = 2.461, p = 0.087; L/L: MD = 0.896, p = 0.958), with ESWT showing significant superiority in U/L at 18 weeks (MD = 3.611, p = 0.010). For SS‐QOL and 10MWT, differences were non‐significant at 6 weeks (SS‐QOL: MD = 4.270, p = 0.714; 10MWT: MD = −1.561, p = 0.115) but slightly greater or superior with ESWT at 18 weeks (SS‐QOL: MD = 4.605, p = 0.209; 10MWT: MD = −1.815, p = 0.020).

**Conclusion:**

Both ESWT and Acu‐TENS with physiotherapy reduced spasticity and improved motor function, mobility, and SS‐QOL in post‐stroke patients. ESWT showed slightly greater gains, but differences were mostly not significant, indicating both are useful additions to physiotherapy.

**Trial Registration:**

CTRI/2025/05/087383

## Graphical abstract

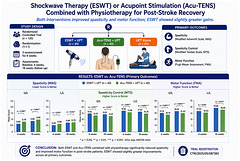

**Abbreviations**
10MWT: 10 Meter Walk Test
Acu‐TENS: Acupoint‐Based Transcutaneous Electrical Nerve Stimulation
ESWT: Extracorporeal Shockwave Therapy
FMA: Fugl‐Meyer Assessment
MAS: Modified Ashworth Scale
MTS: Modified Tardieu Scale
SS‐QOL: Stroke‐Specific Quality of Life
UPT: Usual Physiotherapy Treatment

## 1 Introduction

Stroke is a major contributor to long‐term neurological impairment and is the second leading cause of mortality worldwide (Senarath et al. 2023). One of the leading causes of unexpected death or disability is stroke, which includes both ischemic and hemorrhagic stroke (Zhu et al. 2024). Post‐stroke spasticity is a frequent consequence, with rates of 24.5% at 6 days, 26.7% at 6 weeks, and around 38% in the first year following stroke (Wissel et al. 2010; Watkins et al. 2002). Spasticity, also known as hypertonia, is characterized by increased muscle tone and exaggerated tendon reflexes resulting from hyperexcitability of the stretch reflex due to an imbalance between supraspinal inhibitory and excitatory inputs (Lance 1980). Although spasticity may provide limited functional benefits in some individuals, it is predominantly considered an impairment that compromises motor control and functional performance (Hsu et al. 2021). Early management is therefore essential to prevent long‐term complications such as joint contractures, pressure ulcers, muscle weakness, and pain, which restrict daily activities and community participation (Francisco et al. 2021).

After a stroke, common management approaches for spastic hemiplegia include oral antispasmodic medications (Wagstaff and Bryson 1997), intramuscular injections of botulinum toxin (Jia et al. 2020), and surgical interventions (Zheng et al. 2017). While the benefits of muscle relaxants in lowering resistance to passive movement have been established, it is difficult to avoid their contradictory effects on improving active functional ability (Gracies et al. 2015). Additionally, the potential long‐term adverse effects associated with antispasmodic medications and botulinum toxin—including muscle weakness, sensory impairment, possible dependency, and hepatotoxicity—together with the inherent risks of surgical procedures, warrant careful consideration (Kudva et al. 2021; Camões‐ Barbosa et al. 2020). As the population ages, many nations, particularly Bangladesh, may see a significant rise in the number of elderly stroke patients who require complex medical care, increasing the burden of disease and care (Qian et al. 2023). This is because the prevalence rates of stroke rise rapidly with age and are typically linked to worse outcomes. Thus, physicians, patients, and their families want safer, more effective treatment alternatives that can help patients regain their optimal functional abilities.

Extracorporeal shock wave therapy (ESWT) has emerged as a promising non‐invasive intervention for post‐stroke spasticity and motor recovery. Radial ESWT delivers mechanically generated pressure waves that transfer energy through the skin to underlying tissues. (Walewicz et al. 2019) Its therapeutic effects have been associated with improved muscle extensibility and elasticity (Gao et al. 2019; Stecco et al. 2014), increased nitric oxide production affecting neuromuscular junctions and inflammatory regulation (El‐Shamy et al. 2014; Martínez et al. 2020; Wu et al. 2018), and enhanced angiogenesis and tissue perfusion (Mihai et al. 2022). Recent meta‐analyses have demonstrated that radial ESWT effectively reduces spasticity and improves motor function in both the upper and lower limbs (Cabanas‐Valdés et al. 2020; Cabanas‐Valdés et al. 2019).

Acupuncture and similar treatments have also shown beneficial effects in individuals with post‐stroke spastic hemiplegia. Due to the synergistic effect of acupoint combinations, employing multiple acupoints in a single treatment session often yields more effective results than using a single point. The non‐invasive acupuncture‐related technique known as acupoint‐based TENS (Acu‐TENS) or transcutaneous electrical acupoint stimulation (TEAS) involves applying transcutaneous electrical nerve stimulation (TENS) to specific acupoints (Qian et al. 2023). For many years, TENS has been recommended for treating spasticity due to its affordability, ease of use, and minimal side effects (Fernández‐Tenorio et al. 2018). Acu‐TENS is believed to have the same, or even greater, therapeutic properties as acupuncture, thereby restoring the body's energy balance and stimulating signal transmission (Li et al. 2014).

The effectiveness of radial ESWT and Acu‐TENS methods has been assessed independently in numerous prior studies; however, a direct comparison of their efficacy has not yet been conducted. However, previous research has examined the effectiveness of radial ESWT compared with TENS for upper‐limb spasticity (Senarath et al. 2023). Additionally, acupoint‐based TENS has been investigated in conjunction with transcranial direct current stimulation (tDCS) for the treatment of spasticity (Qian et al. 2023). However, no randomized controlled trial has compared radial ESWT and Acu‐TENS, both combined with usual physiotherapy treatment (UPT), for improving upper‐ and lower‐limb spasticity and motor function after stroke.

Therefore, this study aimed to address this evidence gap by directly comparing the effects of radial ESWT and Acu‐TENS, each combined with UPT, on spasticity and motor function during post‐stroke recovery. We hypothesized that radial ESWT or Acu‐TENS, combined with UPT, would yield greater improvements in spasticity and motor function at post‐intervention and follow‐up assessments.

## 2 Methods

### 2.1 Study Design

**FIGURE 1:**
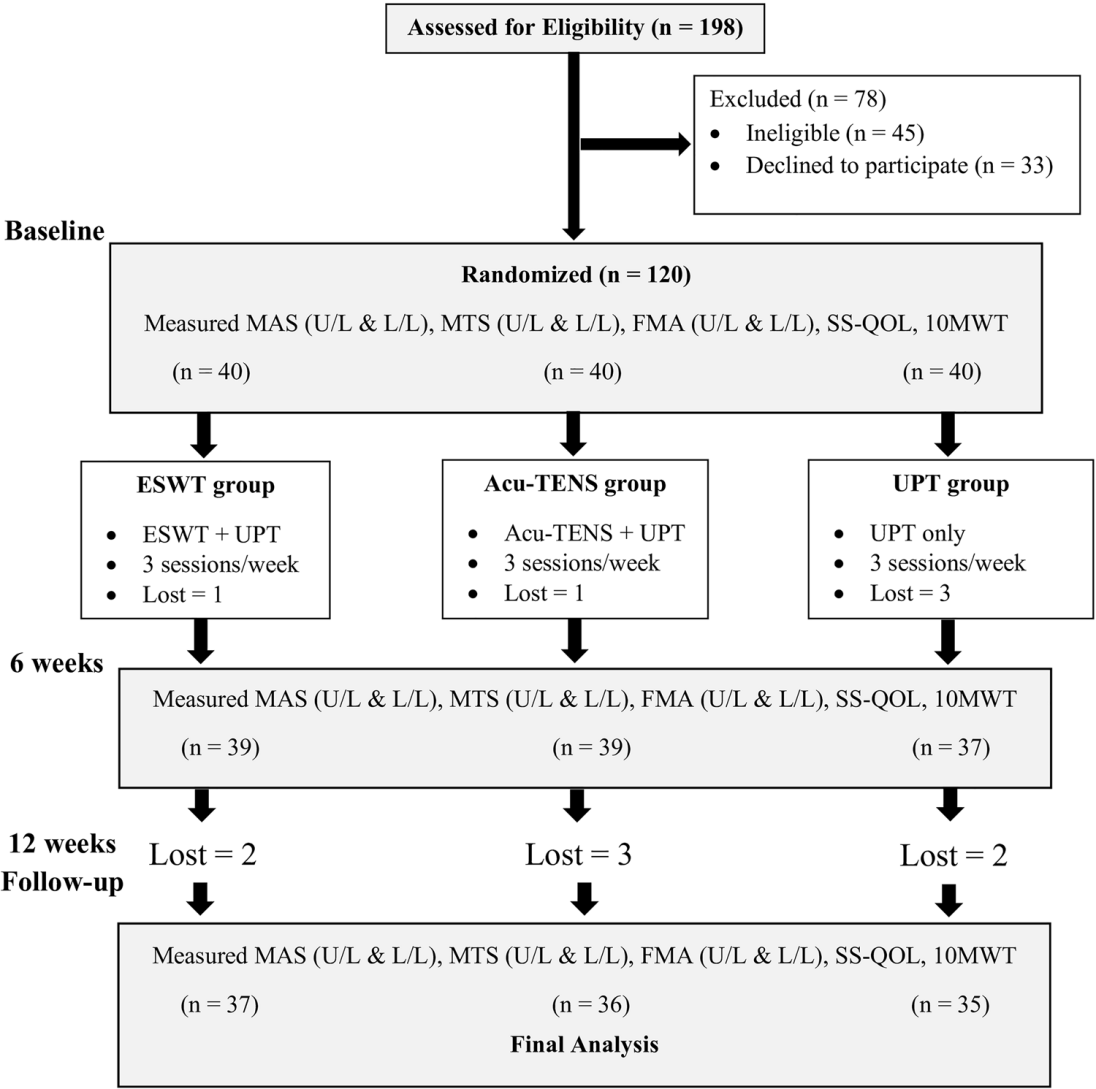
Study flow diagram.

This multicenter, assessor‐blinded randomized controlled trial was conducted in Bangladesh from June 2025 to March 2026. Participants were assigned to three groups (*n* = 40 each): (1) radial ESWT with UPT, (2) Acu‐TENS with UPT, and (3) UPT alone (control group). All participants received a 6‐week intervention program consisting of three sessions per week. Outcomes were measured at three time points: baseline, after 6 weeks, and after 18 weeks, following a 12‐week follow‐up. The study was ethically approved and registered with the Clinical Trials Registry of India (ID: CTRI/2025/05/087383). The results were reported transparently, in accordance with the Consolidated Standards of Reporting Trials (CONSORT) guidelines (Figure 1).

### 2.2 Sample Size Calculation

The sample size was determined using data from a previous rehabilitation intervention involving individuals with stroke, which showed a Cohen's d of 0.50 for spasticity, as measured by the Modified Ashworth Scale (MAS). Assuming equal variances, a baseline mean MAS score of 2, a standard deviation of 0.8, α = 0.05, and 80% power, we calculated that 34 patients per group would be needed (Suputtitada et al. 2024). To account for potential attrition of 15%–20%, we recruited 40 participants per group, resulting in a total sample size of 120. After reaching our target, participant recruitment was stopped.

### 2.3 Participants' Recruitment and Settings

A total of 120 post‐stroke participants were recruited from three study settings: the Physiotherapy and Rehabilitation Department at JUST; SP physiotherapy care; and the Stroke Unit at the Center for the Rehabilitation of the Paralyzed (CRP) in Bangladesh, using simple random sampling to ensure a representative selection. Eligibility screening was conducted by three research assistants who were independent of treatment delivery and outcome assessment. All participants provided written informed consent, which included detailed information about the study's objectives, procedures, potential risks, and benefits, and was obtained using a standardized form approved by the institutional ethics review board. All study documents, including consent forms and personal files, were securely stored in locked cabinets and electronic systems to prevent unauthorized access and maintain data integrity.

### 2.4 Eligibility Criteria

Participants were selected based on predefined study inclusion and exclusion criteria. Participants were included if—both male and female, aged 40–70 with ischemic stroke (confirmed by CT or MRI) lasting over one month; unilateral limb paralysis (Qian et al. 2023); upper‐limb and lower‐limb motor dysfunction; and spasticity (MAS ≥ 1 and ≤ 2), participants must be stable, aware, and free from hearing or cognitive impairments that prevent answering questions; able to walk independently 10 meters (Qian et al. 2023; Ali et al. 2024). Participants were excluded if—serious internal diseases, tumors, infections, or mental illnesses; contraindications for electrical stimulation and ESWT (e.g., pacemakers and implantable devices); a history of epilepsy, seizures, head trauma, or spinal cord surgery; bleeding or clinically significant coagulation disorders, including those receiving anticoagulant therapy (e.g., warfarin or direct oral anticoagulants) or requiring routine coagulation monitoring (e.g., INR assessment); pregnant, lactating, or planning pregnancy within 6 months; skin injuries or scarring at stimulation sites; sensory disturbances or electrical stimulation allergies; recent use of muscle relaxants or botulinum toxin injections in spastic limbs within 6 months (Qian et al. 2023; Ali et al. 2024).

### 2.5 Randomization and Blinding

Participants who met the eligibility criteria were randomly assigned to three parallel groups using a computer‐generated sequence prepared by an independent research assistant. Group assignments were kept in opaque, sealed envelopes and only revealed after baseline assessments to prevent selection bias. The primary investigator supervised the randomization process for monitoring but did not participate in participant recruitment or assignment. Outcome assessors and data analysts remained blinded to group allocations throughout the study, in accordance with standardized procedures. Participants and treating health professionals knew their assigned intervention due to the nature of the programs; however, the study hypotheses and expected group advantages were not disclosed to reduce performance bias among participants. Independent trial monitoring staff ensured compliance with randomization, allocation concealment, and blinding procedures throughout the study.

### 2.6 Study Procedure

Participants were recruited from three study sites following both online and offline advertisements. Eligible participants were first given written informed consent and then randomly assigned to treatment groups. However, the group assignment was only revealed after baseline assessments. All participants completed their assigned interventions over six weeks, with three supervised sessions each week. Participants were instructed to maintain stable medication regimens throughout the study, and no initiation or dosage adjustment of oral antispastic medications occurred during the intervention or follow‐up periods. Outcome measures were collected at baseline, post‐intervention, and follow‐up by blinded, trained assessors. Standardized procedures and internationally recognized guidelines were followed for all assessments and interventions, ensuring consistency, reliability, and methodological rigor. The study adhered to the CONSORT statement for randomized controlled trials, with all procedures documented to promote transparency, reproducibility, and objective measurement of outcomes.

### 2.7 Interventions

All participants received 18 intervention sessions, three each week over 6 weeks. Three supervised sessions per week were selected based on established stroke rehabilitation practice and previous clinical trials, providing an appropriate therapeutic dose while minimizing participant fatigue. UPT was standardized across all groups, while radial ESWT and Acu‐TENS were delivered concurrently in their respective intervention groups to isolate their adjunctive effects. All interventions were delivered by six experienced physiotherapists at the study sites. Target muscles were identified using standardized anatomical landmarks and palpation by the treating physiotherapists. Intervention details are provided below and summarized in Table 1.

**TABLE 1:** Intervention details.

Intervention	Details
Radial ESWT	• Frequency: 5 Hz • Energy flux density: 0.030 mJ/mm^2^ • Dose: 1500 shocks per targeted muscle • Target area: Affected upper and lower limb muscle bellies (midpoint) • Session duration: 30min
Acu‐TENS	• Frequency: 100 Hz • Waveform: Square wave, pulse width 0.2 ms • Intensity: 2–3× sensory threshold (no visible muscle contraction) • Electrode size: 40 mm × 40 mm • Acupoints: 4 WHO‐standard points on affected upper and lower limb • Session duration: 30 min
UPT (Stroke‐specific physiotherapy)	• Flexibility training: Stretching of spastic muscles (upper/lower limb) to reduce tone and prevent contracture • ROM training: Active‐assisted and active range of motion exercises for hemiparetic limbs • Strengthening: Task‐oriented progressive resistance training for affected muscle groups • Trunk control: Bridging, weight‐shifting, and sitting balance exercises • Functional mobility: Sit‐to‐stand training, bed mobility, and transfer training • Balance training: Static and dynamic balance exercises in sitting and standing • Gait training: Overground walking, step training, and weight‐bearing symmetry practice • Task‐specific training: Functional ADL‐oriented activities (reaching, grasping, dressing simulation) • Session duration: 40–60 min Exercise intensity, progression, and repetition were individualized according to each participant's functional status, tolerance, and rehabilitation goals.

All interventions were administered three sessions per week for six weeks (18 sessions in total) by experienced physiotherapists in accordance with standardized study protocols.

Abbreviations: AAROM, Active‐Assisted Range of Motion; Acu‐TENS, Acupuncture‐based Transcutaneous Electrical Nerve Stimulation; ADLs, Activities of Daily Living; ESWT, Extracorporeal Shockwave Therapy; Hz, Hertz; mJ/mm^2^, Millijoules per Square Millimeter; UPT, Usual Physiotherapy Treatment; WHO, World Health Organization.

#### 2.7.1 Experimental Group I: Radial ESWT+UPT (ESWT Group)

Participants in this group received radial ESWT combined with UPT, administered three times weekly for 6 weeks, for a total of 18 sessions. Radial ESWT was delivered using a radial shockwave device (Astar Impactis M/Impactis M+) with a 15 mm applicator tip, operating at a 5 Hz frequency, 1500 shocks per targeted muscle, and an energy level of 0.030 mJ/mm^2^. Treatment was applied to predefined spastic muscle groups commonly affected in post‐stroke flexor synergy patterns, including upper‐limb muscles (biceps brachii, flexor carpi radialis, flexor carpi ulnaris, flexor digitorum superficialis, and flexor digitorum profundus) and lower‐limb muscles (gastrocnemius, soleus, tibialis posterior, and hamstrings), with additional muscles targeted where clinically indicated based on individual spasticity distribution. Shockwaves were delivered perpendicular to the skin over the midpoint of each muscle belly using a coupling gel to ensure optimal energy transmission. Participants were positioned in a relaxed, supported supine or prone posture depending on the treatment area to ensure comfort and consistent application.

#### 2.7.2 Experimental Group II: Acu‐TENS+UPT (Acu‐TENS Group)

Participants in this group underwent UPT three times a week for 6 weeks, in addition to Acu‐TENS. Acu‐TENS was applied for 30 min per session at 100 Hz using a dual‐channel TENS machine (QL/T‐IIA, Sichuan Qianli Beoka Medical Technology Co., Ltd., China) (Qian et al. 2023). After skin cleansing, 40 mm × 40 mm electrodes were applied to four standardized acupoints (Table 2) on the affected upper and lower limbs in all participants. A square wave with a pulse width of 0.2 ms was used for stimulation, and the intensity was adjusted to two to three times the sensory threshold, without causing any noticeable muscle contractions (Qian et al. 2023; Lim 2009).

**TABLE 2:** Locations of acupoints [WHO (14, 28)].

Acupoints	Side	Locations
Quchi (LI 11)	Affected upper limb side	On the lateral midpoint between the depression just lateral to the biceps brachii tendon and the lateral epicondyle of the humerus.
Waiguan (TE 5)	Affected upper limb side	On the posterior forearm, at the midpoint of the interosseous space between the radius and ulna, located 2 cun proximal to the dorsal wrist crease.
Yanglingquan (GB 34)	Affected lower limb side	On the fibular side of the leg, in the depression just in front and below the head of the fibula.
Shangjuxu (ST 37)	Affected lower limb side	On the front of the leg, 6 cm below the lateral depression of the patellar ligament at the knee is located.

#### 2.7.3 Control Group: UPT

All participants in the control group received Usual Physiotherapy Treatment (UPT) three times per week for 6 weeks, for a total of 18 sessions. The UPT program was structured to address multiple aspects of post‐stroke rehabilitation and included stretching exercises to maintain muscle flexibility, active‐assisted range of motion (AAROM) exercises to improve joint mobility, trunk control exercises to enhance core stability, sit‐to‐stand training to promote functional independence, balance and gait training to improve postural control and mobility, and task‐specific functional movements aimed at restoring daily living activities (Liao et al. 2025).

### 2.8 Intervention Fidelity and Adherence Monitoring

All sessions for both groups were video‐recorded, and 25% of the sessions were randomly selected for review by an independent investigator using standardized checklists to assess protocol adherence, intervention technique, and interventionist competence. Before each treatment session, participants were screened for new contraindications, including skin lesions, acute infections, bleeding symptoms, changes in medication, and any new medical conditions that could affect the safety of the intervention. Participant adherence was tracked through session attendance logs for all participants in both groups. The number, length, and form of interventions were monitored using a checklist. This method ensured precise monitoring of intervention delivery and participant compliance without overlap or redundancy.

### 2.9 Outcome Measurements

The primary and secondary outcomes were measured at baseline, post‐intervention, and follow‐up after participants completed 6 weeks of interventions, as assessed by six blinded, trained assessors under the supervision of responsible health personnel.

#### 2.9.1 Primary Outcomes

Spasticity (Modified Ashworth Scale, MAS): Upper‐limb (U/L) and Lower‐limb (L/L) muscle tone was assessed using the MAS. Scores were converted to a 0–5 scale for analysis, and the total MAS score was calculated by summing the average values for U/L‐ and L/L‐specific joints. Standardized training was provided to assessors to ensure consistent passive movement during testing; ICC = 0.64 – 0.87 (Vidmar et al. 2023).

Spasticity control (Modified Tardieu Scale, MTS): Spasticity control in the U/L and L/L was measured using the MTS by recording R1 and R2 angles with a goniometer at different stretch velocities (V1–V3). Muscle response was graded on a 0–5 scale, with higher scores indicating greater resistance; ICC = 0.70 – 0.99 (Ben‐Shabat et al. 2013).

Motor function (Fugl‐Meyer Assessment, FMA: FMA‐U/L and LE): Motor function of the U/L and L/L was assessed using the FMA, a validated and widely used scale for evaluating post‐stroke motor function. The FMA‐U/L includes 33 items assessing the shoulder, elbow, forearm, wrist, and hand, while the FMA‐L/L comprises 17 items evaluating the hip, knee, and ankle. Each item is scored on a 3‐point scale (0 = cannot perform, 1 = partial performance, 2 = full performance), with higher scores indicating better motor function; ICC = 0.85 – 0.99 (Hernández et al. 2020).

#### 2.9.2 Secondary Outcomes

Quality of Life (Stroke‐Specific QoL, SS‐QOL): Stroke‐related quality of life was assessed using the SS‐QOL, which measures patients’ perceived quality of life and functional independence; ICC = 0.80–0.98 (Muus et al. 2007).

Functional mobility (10‐Meter Walk Test, 10MWT): Functional mobility was assessed using the 10MWT, in which participants walked 10 meters at a comfortable, self‐selected pace. The time taken was recorded over three trials, and the average was calculated to evaluate changes in walking ability; ICC = 0.76–0.97 (Scivoletto et al. 2011).

### 2.10 Statistical Analysis

Data analyses were performed using IBM SPSS Statistics version 26 by an independent, blinded statistician. Analyses followed the intention‐to‐treat principle, including all 120 randomized participants. Missing data were handled using multiple imputation under the missing‐at‐random assumption. Twenty datasets were imputed using fully conditional specification, including treatment group, baseline covariates, and all repeated outcome measures. Estimates were pooled using Rubin's rules. Continuous variables were presented as mean ± standard deviation (SD) and categorical variables as frequency and percentages. Baseline sociodemographic differences among the ESWT, Acu‐TENS, and UPT groups were assessed using one‐way ANOVA for continuous variables and chi‐square tests for categorical variables. For all outcome measures, group differences at baseline, 6 weeks, and 18 weeks were evaluated with one‐way ANOVA. Within‐group changes from baseline to 6 weeks, from baseline to 18 weeks, and from 6 to 18 weeks were analyzed using mixed change‐score comparisons, with results expressed as mean change ± standard deviation. Between‐group effects were assessed by comparing change scores with group × time contrasts, reported as mean differences with 95% confidence intervals for ESWT versus Acu‐TENS, ESWT versus UPT, and Acu‐TENS versus UPT. Repeated‐measures ANOVA was used to assess within‐group changes over time, between‐group differences, and time‐by‐group interactions, with results reported as *F*‐value, *p*‐value, and partial eta squared (ηp^2^) as a measure of effect size. Bonferroni post hoc pairwise comparisons of outcome measures are also reported in Supplementary Material 2. Statistical significance was defined as ***p < 0.05, **p < 0.01, and *p < 0.001.

### 2.11 Ethical Considerations

All procedures were conducted in accordance with the 2020 Declaration of Helsinki and the institutional ethical guidelines. The study was approved by the Institutional Review Board of the Department of Physiotherapy and Rehabilitation at Jashore University of Science and Technology (Approval ID: PTR‐JUST/IRB/2025/05/192404) and was registered in the Clinical Trials Registry‐India (CTRI/2025/05/087383). Before enrollment, written informed consent was obtained from all participants and their guardians.

### 2.12 Participants and Public Involvement

Participants or the public were not involved in the design, conduct, or reporting of the trial.

## 3 Results

### 3.1 Participant Flow and Baseline Characteristics

A total of 120 participants were enrolled and equally allocated to three intervention groups. Among them, 115 completed the post‐intervention assessment, and 108 completed the follow‐up evaluation. Participant withdrawals were primarily attributable to illness and family‐related reasons, as illustrated in Figure 1. Baseline demographic and clinical characteristics are presented in Table 3. No statistically significant differences were observed among the three groups for any demographic or clinical variable (all *p* > 0.05), confirming successful randomization and baseline comparability. Participants were comparable with respect to age, sex distribution, body mass index, duration of stroke, stroke characteristics, smoking history, and family history of stroke. These findings indicate that the treatment groups were well balanced before intervention and that subsequent between‐group differences are unlikely to be attributable to baseline imbalances.

**TABLE 3:** Baseline characteristics.

Characteristics	Patient groups			*p*‐value
	ESWT group (*n* = 40)	Acu‐TENS group (*n* = 40)	UPT group (*n* = 40)	
Age, mean (SD), yrs	55.70 (6.21)	57.25 (7.65)	56.45 (6.50)	0.597^a^
Gender, No. (%)				
Male	17 (42.5%)	13 (32.5%)	21 (52.5%)	0.197^b^
Female	23 (57.5%)	27 (67.5%)	19 (47.5%)	
Body mass index (BMI), mean (SD)	26.44 (2.69)	26.72 (2.79)	27.21 (2.29)	0.405^a^
Duration of Stroke, mean (SD), months	5.08 (2.48)	5.25 (2.51)	5.40 (2.20)	0.833^a^
Cerebral infarcts, No. (%)				
Single	28 (70.0%)	25 (62.5%)	31 (77.5%)	0.346^b^
Multiple	12 (30.0%)	15 (37.5%)	9 (22.5%)	
Smoking history, No. (%)				
Yes	30 (75.0%)	27 (67.5%)	28 (70.0%)	0.756^b^
No	10 (25.0%)	13 (32.5%)	12 (30.0%)	
Family history, No. (%)				
Yes	23 (57.5%)	25 (62.5%)	21 (52.5%)	0.666^b^
No	17(42.5%)	15 (37.5%)	19 (47.5%)	

^a^ One‐way ANOVA test; ^b^Chi‐square test.

Abbreviations: Acu‐TENS, Acupoint‐based Transcutaneous Electrical Nerve Stimulation; ESWT. Extracorporeal Shockwave Therapy; UPT. Usual Physiotherapy Treatment.

### 3.2 Comparison of Outcome Measurements

Participants across all three groups—ESWT, Acu‐TENS, and UPT—demonstrated significant improvements in all measured outcomes over time (*p* < 0.05). Nevertheless, the ESWT group showed slightly greater improvements across multiple outcomes than the Acu‐TENS and UPT groups at post‐test and follow‐up. A comprehensive overview of all outcome measurements is provided below:

#### 3.2.1 Primary Outcomes

##### 3.2.1.1 Spasticity (MAS and MTS)

Repeated‐measures ANOVA demonstrated a significant group × time interaction for upper‐limb MAS (*F* = 7.814, *p* < 0.001, ηp^2^ = 0.118), indicating that the magnitude of change differed among the three interventions. However, no significant interaction was observed for lower‐limb MAS (*F* = 2.081, p = 0.084, ηp^2^ = 0.034), despite significant improvements over time across all groups.

Between‐group analyses demonstrated a gradient of improvement, with the largest reduction in upper‐limb spasticity observed in the ESWT group, followed by the Acu‐TENS and UPT groups. Compared with UPT, ESWT produced significantly greater reductions in upper‐limb MAS scores at both the post‐intervention (MD = −0.73, 95% CI −1.05 to −0.40) and follow‐up (MD = −0.70, 95% CI −1.02 to −0.38) periods. Acu‐TENS also demonstrated greater improvement than UPT at both assessment points (6 weeks: MD = −0.40, 95% CI −0.70 to −0.10; 18 weeks: MD = −0.43, 95% CI −0.72 to −0.13). Differences between ESWT and Acu‐TENS were comparatively small, and their confidence intervals included the null value, indicating no statistically significant difference between these two active interventions.

For the MTS, all groups showed improvements in spasticity control over time. A significant group × time interaction was identified for lower‐limb MTS (*F* = 3.028, p = 0.018, ηp^2^ = 0.049), whereas the interaction for upper‐limb MTS was not significant (*F* = 1.870, p = 0.117, ηp^2^ = 0.031). Between‐group comparisons demonstrated that ESWT achieved greater improvements than UPT in lower‐limb MTS at follow‐up (MD = 2.40°, 95% CI 0.70–4.09), while Acu‐TENS also demonstrated significantly greater improvement than UPT (MD = 1.92°, 95% CI 0.15–3.69). Differences between ESWT and Acu‐TENS were relatively modest, with confidence intervals crossing zero. For upper‐limb MTS, between‐group differences were generally smaller and consistent with the absence of a significant interaction effect.

##### 3.2.1.2 Motor Function (FMA)

Repeated‐measures ANOVA demonstrated significant group × time interactions for both upper‐limb FMA (*F* = 10.788, *p* < 0.001, ηp^2^ = 0.156) and lower‐limb FMA (*F* = 3.705, p = 0.006, ηp^2^ = 0.060), indicating that the magnitude of motor recovery differed among the interventions over time.

For upper‐limb motor function, improvements were observed across all groups, with the greatest gains in the ESWT group, followed by the Acu‐TENS and UPT groups. Between‐group comparisons demonstrated significantly greater improvement with ESWT than UPT at both post‐intervention (MD = 6.11, 95% CI 3.31–8.91) and follow‐up (MD = 8.07, 95% CI 5.36–10.77). Acu‐TENS also achieved significantly greater improvement than UPT at post‐intervention (MD = 3.52, 95% CI 0.94–6.09) and follow‐up (MD = 4.32, 95% CI 1.48–7.17). Differences between ESWT and Acu‐TENS were comparatively smaller and reached statistical significance only at follow‐up (MD = 3.74, 95% CI 0.85–6.64).

A similar pattern was observed for lower‐limb motor function, with progressive improvements across all three groups. ESWT demonstrated significantly greater improvement than UPT at post‐intervention (MD = 2.70, 95% CI 0.79–4.61) and follow‐up (MD = 3.14, 95% CI 1.24–5.03). Acu‐TENS also showed greater improvement than UPT at both assessment points (6 weeks: MD = 2.30, 95% CI 0.14–4.47; 18 weeks: MD = 2.43, 95% CI 0.26–4.61), whereas differences between ESWT and Acu‐TENS remained small and were not statistically significant.

#### 3.2.2 Secondary Outcomes

##### 3.2.2.1 Quality of Life (SS‐QOL)

Repeated‐measures ANOVA demonstrated a significant group × time interaction (*F* = 4.276, p = 0.002, ηp^2^ = 0.068), indicating differential improvement among the interventions.

Between‐group analyses demonstrated that the greatest improvement in SS‐QOL was observed in the ESWT group, followed by the Acu‐TENS and UPT groups. Compared with UPT, ESWT produced significantly greater improvement at post‐intervention (MD = 11.64, 95% CI 3.03–20.25) and follow‐up (MD = 11.61, 95% CI 4.81–18.42). Acu‐TENS also demonstrated significantly greater improvement than UPT at follow‐up (MD = 3.76, 95% CI 1.04–10.59), whereas differences between ESWT and Acu‐TENS were comparatively smaller and not statistically significant at either assessment point.

##### 3.2.2.2 Functional Mobility (10MWT)

Repeated‐measures ANOVA demonstrated a significant main effect of time, indicating progressive improvement throughout the intervention period; however, the group × time interaction was not statistically significant (*F* = 1.946, p = 0.104, ηp^2^ = 0.032), suggesting that the temporal pattern of improvement was broadly comparable across the three interventions.

Although the interaction effect was not significant, between‐group comparisons indicated a tendency toward greater improvement in the active intervention groups. Compared with UPT, ESWT demonstrated greater reductions in walking time at follow‐up (MD = −2.50 s, 95% CI −5.48 to 0.48), whereas Acu‐TENS showed a smaller between‐group difference (MD = 0.26 s, 95% CI, −2.72 to 3.24). Direct comparisons between ESWT and Acu‐TENS demonstrated only modest differences (MD = −2.76 s, 95% CI −5.18 to −0.34). Collectively, these findings suggest that all three interventions contributed to improved walking performance, although differences in the rate of improvement among groups were relatively small.

Tables 4, 5, 6 and Supplementary Materials 1–2 present the detailed values for all outcome measures across the three assessment timelines. In addition, Supplementary Material 3 includes figures illustrating the corresponding findings and depicting changes in outcome measures over time.

**TABLE 4:** Mean (SD) scores of all outcome measures.

Outcome measures	Patient groups			*p‐*value
	ESWT group (*n* = 40)	Acu‐TENS group (*n* = 40)	UPT group (*n* = 40)	
MAS—U/L				
Baseline	1.95 (0.55)	1.80 (0.61)	1.68 (0.57)	0.108
6 weeks	0.60 (0.71)	0.78 (0.58)	1.05 (0.55)	0.006**
18 weeks	0.83 (0.68)	0.95 (0.55)	1.25 (0.49)	0.004**
MAS—L/L				
Baseline	1.55 (0.56)	1.43 (0.71)	1.53 (0.55)	0.627
6 weeks	0.50 (0.68)	0.58 (0.59)	0.85 (0.48)	0.023*
18 weeks	0.68 (0.66)	0.70 (0.56)	0.98 (0.53)	0.043*
MTS (R2 ‐ R1 angle)—U/L				
Baseline	12.27 (2.05)	11.93 (2.62)	11.15 (2.11)	0.082
6 weeks	15.82 (1.74)	14.24 (2.96)	13.88 (2.19)	0.001**
18 weeks	15.05 (1.84)	13.63 (2.88)	13.18 (2.15)	0.001**
MTS (R2 ‐ R1 angle)—L/L				
Baseline	14.15 (2.69)	13.13 (2.57)	12.95 (2.15)	0.069
6 weeks	18.58 (3.10)	17.08 (2.94)	15.95 (3.64)	0.002**
18 weeks	17.83 (2.71)	16.33 (2.89)	14.23 (3.33)	0.0001***
FMA—U/L				
Baseline	17.82 (2.90)	17.95 (3.31)	18.66 (3.00)	0.426
6 weeks	31.24 (5.79)	28.78 (4.79)	25.97 (4.22)	0.0001***
18 weeks	35.04 (5.04)	31.43 (5.53)	27.81 (5.59)	0.0001***
FMA—L/L				
Baseline	9.32 (1.44)	8.82 (2.17)	9.72 (1.90)	0.101
6 weeks	17.53 (3.56)	16.63 (4.12)	15.23 (4.30)	0.039*
18 weeks	19.85 (3.70)	18.65 (4.39)	17.12 (4.20)	0.014*
SS‐QOL				
Baseline	55.25 (12.69)	58.50 (9.59)	54.69 (8.97)	0.222
6 weeks	78.36 (13.37)	74.09 (15.52)	66.15 (18.92)	0.004**
18 weeks	81.93 (8.87)	77.32 (11.73)	69.75 (12.79)	0.0001***
10MWT (seconds)				
Baseline	14.25 (4.36)	15.47 (7.49)	13.30 (4.95)	0.247
6 weeks	8.28 (3.65)	11.51 (3.83)	9.84 (2.33)	0.0001***
18 weeks	7.35 (2.69)	11.07 (3.62)	9.17 (2.34)	0.0001***

One‐way ANOVA test.

***p < 0.05, **p < 0.01, and *p < 0.001.

Abbreviations: 10MWT, 10‐Meter Walk Test; Acu‐TENS, Acupoint‐based Transcutaneous Electrical Nerve Stimulation; ESWT, Extracorporeal Shockwave Therapy; FMA, Fugl‐Meyer Assessment; MAS, Modified Ashworth Scale; MTS, Modified Tardieu Scale; SS‐QOL, Stroke‐Specific Quality of Life; UPT, Usual Physiotherapy Treatment.

**TABLE 5:** Mixed between‐group analysis—MD (95% CI).

ESWT—Acu‐TENS			
Outcome measures	6 weeks – Baseline	18 weeks – Baseline	18 weeks – 6 weeks
MAS—U/L	−0.33 (‐0.67 to 0.02)	−0.28 (‐0.61 to 0.06)	0.05 (‐0.21 to 0.31)
MAS—L/L	−0.20 (‐0.55 to 0.15)	−0.15 (‐0.45 to 0.15)	0.05 (‐0.11 to 0.21)
MTS (R2 ‐ R1 angle)—U/L	1.23 (0.08 to 2.38)	1.07 (‐0.14 to 2.28)	−0.17 (‐0.68 to 0.35)
MTS (R2 ‐ R1 angle)—L/L	0.47 (‐1.22 to 2.17)	0.47 (‐1.17 to 2.12)	0.00 (‐0.73 to 0.73)
FMA—U/L	2.59 (‐0.32 to 5.51)	3.74 (0.85 to 6.64)	1.15 (‐0.78 to 3.08)
FMA—L/L	0.40 (‐1.55 to 2.34)	0.70 (‐1.33 to 2.74)	0.30 (‐1.29 to 1.89)
SS‐QOL	7.52 (‐0.22 to 15.26)	7.85 (1.04 to 14.66)	0.34 (‐2.78 to 3.45)
10MWT (seconds)	−2.51 (‐5.14 to 0.12)	−2.76 (‐5.18 to ‐0.34)	−0.25 (‐1.03 to 0.52)
ESWT—UPT			
MAS—U/L	−0.73 (‐1.05 to ‐0.40)	−0.70 (‐1.02 to ‐0.38)	0.03 (‐0.17 to 0.22)
MAS—L/L	−0.38 (‐0.71 to ‐0.04)	−0.33 (‐0.63 to ‐0.02)	0.05 (‐0.14 to 0.24)
MTS (R2 ‐ R1 angle)—U/L	0.82 (‐0.26 to 1.91)	0.75 (‐0.39 to 1.89)	−0.07 (‐0.57 to 0.43)
MTS (R2 ‐ R1 angle)—L/L	1.42 (‐0.22 to 3.06)	2.40 (0.70 to 4.09)	0.98 (0.11 to 1.84)
FMA—U/L	6.11 (3.31 to 8.91)	8.07 (5.36 to 10.77)	1.96 (‐0.20 to 4.12)
FMA—L/L	2.70 (0.79 to 4.61)	3.14 (1.24 to 5.03)	0.43 (‐1.17 to 2.04)
SS‐QOL	11.64 (3.03 to 20.25)	11.61 (4.81 to 18.42)	−0.03 (‐4.00 to 3.95)
10MWT (seconds)	−2.02 (‐5.14 to 1.10)	−2.50 (‐5.48 to 0.48)	−0.48 (‐1.24 to 0.28)
Acu‐TENS—UPT			
MAS—U/L	−0.40 (‐0.70 to ‐0.10)	−0.43 (‐0.72 to ‐0.13)	−0.03 (‐0.27 to 0.22)
MAS—L/L	−0.18 (‐0.50 to 0.15)	−0.18 (‐0.48 to 0.13)	0.00 (‐0.18 to 0.18)
MTS (R2 ‐ R1 angle)—U/L	−0.41 (‐1.59 to 0.76)	−0.32 (‐1.53 to 0.89)	0.09 (‐0.48 to 0.67)
MTS (R2 ‐ R1 angle)—L/L	0.95 (‐0.77 to 2.67)	1.92 (0.15 to 3.69)	0.98 (0.09 to 1.86)
FMA—U/L	3.52 (0.94 to 6.09)	4.32 (1.48 to 7.17)	0.81 (‐1.10 to 2.71)
FMA—L/L	2.30 (0.14 to 4.47)	2.43 (0.26 to 4.61)	0.13 (‐1.17 to 1.44)
SS‐QOL	4.12 (‐4.49 to 12.73)	3.76 (‐3.07 to 10.59)	−0.36 (‐4.39 to 3.66)
10MWT (seconds)	0.49 (‐2.52 to 3.49)	0.26 (‐2.72 to 3.24)	−0.23 (‐0.76 to 0.31)

Abbreviations: 10MWT, 10‐Meter Walk Test, Acu‐TENS, Acupoint‐based Transcutaneous Electrical Nerve Stimulation; ESWT, Extracorporeal Shockwave Therapy; FMA, Fugl‐Meyer Assessment; MAS, Modified Ashworth Scale; MTS, Modified Tardieu Scale; SS‐QOL, Stroke‐Specific Quality of Life; UPT, Usual Physiotherapy Treatment.

**TABLE 6:** Repeated measures ANOVA.

Variables	F p ηp^2^ (Time)	F p ηp^2^ (Group)	F p ηp^2^ (Interaction)
MAS—U/L	F = 153.313 p = 0.0001*** ηp^2^ = 0.567	F = 860.816 p = 0.0001*** ηp^2^ = 0.880	F = 7.814 p = 0.0001*** ηp^2^ = 0.118
MAS—L/L	F = 126.906 p = 0.0001*** ηp^2^ = 0.520	F = 514.492 p = 0.0001*** ηp^2^ = 0.815	F = 2.081 p = 0.084 ηp^2^ = 0.034
MTS (R2 ‐ R1 angle)—U/L	F = 106.619 p = 0.0001*** ηp^2^ = 0.477	F = 5874.128 p = 0.0001*** ηp^2^ = 0.980	F = 1.870 p = 0.117 ηp^2^ = 0.031
MTS (R2 ‐ R1 angle)—L/L	F = 84.788 p = 0.0001*** ηp^2^ = 0.420	F = 5904.322 p = 0.0001*** ηp^2^ = 0.981	F = 3.028 p = 0.018* ηp^2^ = 0.049
FMA—U/L	F = 357.666 p = 0.0001*** ηp^2^ = 0.754	F = 8065.701 p = 0.0001*** ηp^2^ = 0.986	F = 10.788 p = 0.0001*** ηp^2^ = 0.156
FMA—L/L	F = 321.737 p = 0.0001*** ηp^2^ = 0.733	F = 4165.181 p = 0.0001*** ηp^2^ = 0.973	F = 3.705 p = 0.006** ηp^2^ = 0.060
SS‐QOL	F = 128.170 p = 0.0001*** ηp^2^ = 0.523	F = 6049.788 p = 0.0001*** ηp^2^ = 0.981	F = 4.276 p = 0.002** ηp^2^ = 0.068
10MWT (seconds)	F = 65.912 p = 0.0001*** ηp^2^ = 0.360	F = 1823.195 p = 0.0001*** ηp^2^ = 0.940	F = 1.946 p = 0.104 ηp^2^ = 0.032

***p < 0.05, **p < 0.01, and *p < 0.001.

Abbreviations: 10MWT, 10‐Meter Walk Test, Acu‐TENS, Acupoint‐based Transcutaneous Electrical Nerve Stimulation; ESWT, Extracorporeal Shockwave Therapy; FMA, Fugl‐Meyer Assessment; MAS, Modified Ashworth Scale; MTS, Modified Tardieu Scale; SS‐QOL, Stroke‐Specific Quality of Life; UPT, Usual Physiotherapy Treatment.

### 3.3 Adverse Effects Monitoring and Management

During the study, six research assistants and a monitoring team tracked each participant's intervention sessions and any relevant adverse effects or serious consequences. We found that 13 participants reported minor adverse events, including 8 in the experimental groups (ESWT or Acu‐TENS) and 5 in the control group (UPT). In the experimental groups, 5 participants experienced transient muscle soreness and a mild increase in spasticity in the affected limb following the intervention sessions, which resolved within 1–2 days without additional treatment. Three participants reported brief episodes of fatigue and slight dizziness during exercise, which subsided after short rest periods. In the control group, 5 participants experienced temporary stiffness and mild discomfort in the hemiplegic limbs, which improved with rest and minor adjustments in physiotherapy intensity. No serious adverse events occurred, and participants completed the trial without long‐term complications.

## 4 Discussion

This multicenter randomized controlled trial investigated the comparative effectiveness of radial ESWT and Acu‐TENS, each combined with UPT, in reducing spasticity and improving motor function during post‐stroke recovery. All three groups demonstrated significant improvements over time, underscoring the benefits of a structured rehabilitation program. Participants receiving radial ESWT generally showed greater improvements in spasticity, motor recovery, functional mobility, and stroke‐specific quality of life than those receiving Acu‐TENS or UPT alone. However, these findings should be interpreted as indicating a relative advantage rather than consistent superiority of radial ESWT, as between‐group differences were not uniformly significant across all outcomes. Consistent with these findings, repeated‐measures analyses yielded relatively large partial eta squared (ηp^2^) values for several outcomes, indicating that the interventions explained a substantial proportion of the variance in changes over time. As ηp^2^ reflects variance explained within the repeated‐measures model and may be influenced by the correlation structure of repeated observations and low within‐participant variability, these estimates should be interpreted alongside the observed between‐group differences, 95% confidence intervals, and the clinical relevance of the treatment effects rather than as standalone indicators of clinical benefit.

Spasticity remains a major barrier to post‐stroke functional recovery due to increased muscle tone, reflex hyperexcitability, and impaired joint mobility (Wissel et al. 2010; Watkins et al. 2002). In this trial, spasticity outcomes, measured by MAS and MTS, showed significant reductions across all groups; however, improvements were consistently greater in the ESWT group. The repeated‐measures analyses demonstrated significant group × time interaction effects across MAS and MTS outcomes, with partial eta squared (ηp^2^) values ranging from 0.031 to 0.118, indicating that the interventions explained a meaningful proportion of the variance in spasticity changes over time. As partial eta squared quantifies variance explained within the statistical model, these values should be interpreted alongside the observed between‐group differences and their clinical relevance. These findings align with previous randomized trials and systematic reviews reporting that radial ESWT significantly reduces post‐stroke spasticity (Hsu et al. 2021; Mihai et al. 2020; Özbek and Tıkız 2024). The therapeutic effects may be explained by multiple mechanisms. First, radial ESWT may reduce muscle stiffness by mechanically disrupting fibrotic changes and improving viscoelastic properties within the muscle–tendon complex. Second, radial ESWT has been shown to stimulate nitric oxide synthesis and local microcirculation, thereby potentially facilitating neuromuscular relaxation and tissue regeneration. Third, the mechanical impulses generated by radial ESWT may modulate spinal reflex activity and reduce hyperexcitability of the stretch reflex, thereby contributing to long‐term reduction in tone. Previous meta‐analyses have reported significant improvements in MAS scores following radial ESWT interventions in both upper and lower limbs among stroke survivors, supporting the physiological plausibility of these mechanisms (Hsu et al. 2021; Mihai et al. 2020). Acu‐TENS also produced significant improvements in spasticity compared with usual physiotherapy, although effects were smaller than those observed with radial ESWT. Acu‐TENS is believed to influence spasticity primarily by activating sensory afferent fibers at acupuncture points, thereby enhancing inhibitory interneuron activity and reducing motor neuron excitability. Studies examining acupoint‐based electrical stimulation have reported improvements in muscle tone and neuromuscular control, although the overall effect size is generally smaller than that observed with radial ESWT (Qian et al. 2023; Wang et al. 2022). The present study corroborates previous evidence by demonstrating that both radial ESWT and Acu‐TENS effectively reduced spasticity, although the magnitude of improvement was greater following radial ESWT. While treatment effects were largely maintained over the 12‐week follow‐up, both groups exhibited a modest attenuation of benefit, with slight increases in muscle tone relative to post‐intervention values. Nevertheless, spasticity remained significantly lower than at baseline, indicating sustained therapeutic effects beyond the intervention period. This pattern suggests that, although both interventions produce durable neuromuscular benefits, the effects may gradually diminish over time, highlighting the potential value of maintenance strategies or longer treatment duration to optimize long‐term spasticity management. The sustained reduction in spasticity may reflect persistent neuromuscular adaptations, including alterations in muscle mechanical properties and modulation of neural pathways involved in motor control.

Motor function, a central goal of stroke rehabilitation, improves with gains in voluntary movement, directly influencing independence and participation in daily activities (Cabanas‐Valdés et al. 2020). Motor function, assessed using the FMA, improved significantly across all groups, with significant group × time interaction effects and partial eta squared (ηp^2^) values of 0.156 for the upper extremity and 0.060 for the lower extremity, indicating a greater intervention effect on motor recovery. However, the radial ESWT group showed superior improvements in both upper‐ and lower‐limb motor function, with statistically significant advantages over Acu‐TENSUPT and UPT. The improvement in FMA scores observed with radial ESWT may be explained by the combined effects of spasticity reduction and neuromuscular facilitation (Mihai et al. 2020). By reducing abnormal muscle tone, ESWT may enable more efficient motor unit recruitment and improve coordination of voluntary movement. Previous studies also suggest that ESWT can stimulate peripheral nerve regeneration, increase neuromuscular junction activity, and promote neuroplastic changes within the motor cortex (Liu and Zhang 2025). These mechanisms may collectively enhance the recovery of voluntary motor control. Previous clinical studies have reported similar findings, indicating that ESWT can improve motor function in stroke patients when combined with conventional rehabilitation programs. For example, several randomized trials have demonstrated significant improvements in FMA scores and other motor assessments following radial ESWT interventions targeting spastic muscles of the upper and lower limbs (Mihai et al. 2020; Özbek and Tıkız 2024; Liu and Zhang 2025). The present trial strengthens this evidence by directly comparing radial ESWT with another non‐pharmacological modality, Acu‐TENS. Although Acu‐TENS also improved motor function relative to baseline, its effects were smaller than those observed in the radial ESWT group. This difference may be attributable to the distinct mechanisms underlying the two approaches. While Acu‐TENS primarily influences neural excitability through sensory stimulation (Wang et al. 2022; Liu et al. 2024), radial ESWT may produce both neuromuscular and biomechanical changes that more directly facilitate motor recovery (Mihai et al. 2020; Liu and Zhang 2025). Overall, both interventions appear to enhance motor recovery when used as adjuncts to physiotherapy, with radial ESWT demonstrating greater therapeutic benefit than Acu‐TENS.

Quality‐of‐Life outcomes, as measured by the SS‐QOL scale, improved significantly across all groups, indicating that rehabilitation positively influences patient‐centered outcomes beyond changes in impairment level. Participants receiving radial ESWT generally showed greater improvements; however, variability across domains suggests cautious interpretation. Improvements likely reflect combined effects of reduced spasticity, enhanced motor function, and increased independence in daily activities. Previous studies have similarly reported that reductions in spasticity and improvements in mobility are strongly associated with enhanced psychosocial well‐being among stroke survivors (Boehme et al. 2021). Although Acu‐TENS also improved SS‐QOL scores compared with UPT, the magnitude of improvement remained smaller than that observed with radial ESWT. Functional mobility outcomes measured by the 10MWT further support the superiority of radial ESWT as an adjunctive option. Participants receiving radial ESWT demonstrated significantly faster walking speeds compared with those receiving Acu‐TENS or UPT. Improvements in gait performance likely reflect the combined effects of reduced spasticity, improved joint mobility, and enhanced motor coordination. Gait recovery is closely linked to independence and community participation after stroke (Lee et al. 2022). Therefore, the improvements observed in walking ability are likely to have meaningful clinical implications. Enhanced mobility can facilitate greater engagement in rehabilitation activities and daily tasks, potentially accelerating overall recovery. However, between‐group differences were not consistent across all comparisons, and some did not reach statistical significance. Therefore, radial ESWT should be interpreted as a potentially beneficial adjunct rather than a definitive superior intervention for gait recovery. Importantly, all groups demonstrated improvement, reinforcing the fundamental role of structured physiotherapy in functional mobility recovery.

Overall, this multicenter randomized controlled trial provides clinically relevant evidence for optimizing post‐stroke rehabilitation. Radial ESWT, when integrated into physiotherapy, may offer additional benefits for reducing spasticity and improving motor and functional outcomes. However, these effects should be interpreted as additive rather than definitively superior, given variability in statistical significance across outcomes. Acu‐TENS also demonstrated beneficial effects on spasticity, motor function, and quality of life, although with smaller effect magnitudes. This suggests that acupoint‐based electrical stimulation may serve as a complementary neuromodulatory approach, particularly where radial ESWT is unavailable or impractical. Importantly, the improvements observed across all groups underscore the central role of structured physiotherapy interventions in post‐stroke rehabilitation. Conventional physiotherapy approaches—including task‐specific training, balance exercises, gait training, and functional mobility practice—remain the cornerstone of recovery by promoting neuroplasticity, improving motor control, and supporting functional independence. Adjunctive therapies such as ESWT and Acu‐TENS may enhance specific aspects of neuromuscular recovery; however, their effectiveness is likely maximized when integrated within comprehensive physiotherapy programs. Therefore, rehabilitation strategies should prioritize individualized physiotherapy and consider adjunct modalities as complementary tools to support optimal recovery.

### 4.1 Strengths and Limitations

This study has several strengths. It employed a multicenter randomized controlled design with assessor blinding and standardized intervention protocols, enhancing the internal validity of the findings. The inclusion of both upper‐ and lower‐limb outcomes provides a comprehensive evaluation of functional recovery. Furthermore, the use of multiple validated outcome measures and a follow‐up period allowed assessment of both short‐term and sustained treatment effects. Despite these strengths, several limitations should be acknowledged. First, the study was conducted in selected rehabilitation centers in Bangladesh, which may limit the generalizability of the findings to other healthcare contexts or populations. Second, although follow‐up assessments were conducted at 18 weeks, longer‐term outcomes beyond this period remain unknown. Third, blinding of participants and therapists was not feasible due to the nature of the interventions, which may introduce some degree of performance bias. Fourth, target muscles were localized using standardized anatomical landmarks and palpation without musculoskeletal ultrasound guidance, which may have limited targeting precision, although this approach reflects routine clinical practice. Fifth, the study did not include objective neurophysiological assessments, such as electromyography or imaging, to clarify the mechanisms underlying the observed improvements. Sixth, multiple imputation assumes that data were missing at random; if this assumption were violated, some bias may remain. Finally, economic evaluations were not performed, which would be valuable for assessing the cost‐effectiveness of incorporating shockwave therapy into routine rehabilitation practice. Future research should address these limitations by conducting larger multicenter trials with extended follow‐up periods, incorporating neurophysiological measurements to explore underlying mechanisms, and evaluating the cost‐effectiveness of adjunctive therapies in diverse healthcare settings.

## 5 Conclusion

Both radial ESWT and Acu‐TENS, when combined with standard physiotherapy, demonstrated additional benefits in improving spasticity, motor function, mobility, and stroke‐specific quality of life in individuals recovering from stroke. Radial ESWT showed relatively greater improvements in selected outcomes, particularly spasticity and motor function; however, this advantage was not consistent across all measures and should not be interpreted as overall superiority. Acu‐TENS also produced meaningful improvements beyond those achieved with physiotherapy alone, supporting its role as a feasible adjunctive intervention. Overall, these findings support the use of evidence‐based adjunct therapies alongside physiotherapy to enhance post‐stroke rehabilitation outcomes, while further research is needed to confirm comparative effectiveness and long‐term benefits.

## Author Contributions

**Meer Hossain Hero**: investigation, data curation, Writing – review and editing, resources. **Mahomuda Afroze**: supervision, validation, writing – review and editing, visualization. **Saddam Hossain**: methodology, data curation, investigation, formal analysis, writing – review and editing. **Mahdi Ul Bari**: software, visualization, writing – review and editing. **Tofajjal Hossain**: methodology, investigation, visualization, formal analysis, writing – review and editing. **Arnob Datta**: resources, writing – review and editing. **Md. Adnan Ibne Reza**: data curation, software, formal analysis, writing – review and editing. **Kazi Md Azman Hossain**: conceptualization, methodology, project administration, supervision, writing – original draft, funding acquisition, writing – review and editing. **Ahamadullah Hil Galeb**: supervision, validation, writing – review and editing. **Jannatul Ferdous Rikti**: formal analysis, data curation, writing – review and editing.

## Funding

This study was partially funded by the Department of Physiotherapy and Rehabilitation of Jashore University of Science and Technology (Grant No: 25‐FoHS‐04), which provided partial funding for this trial's data collection and intervention. The funding authority had no role in the study design, data collection, analysis, interpretation, or manuscript writing.

## Ethics Statement

The Institutional Review Board of the Department of Physiotherapy and Rehabilitation at Jashore University of Science and Technology approved this study (Approval ID: PTR‐JUST/IRB/2025/05/192404).

## Consent

All study participants provided written informed consent before enrollment.

## Conflicts of Interest

The authors declare no conflicts of interest.

## Supporting information




**Supplementary Materials**: brb371652‐sup‐0001‐SuppMat.docx

**Supplementary Materials**: brb371652‐sup‐0002‐SuppMat.docx

**Supplementary Materials**: brb371652‐sup‐0003‐SuppMat.docx

## Data Availability

The datasets used and/or analyzed in this study are available from the corresponding author upon reasonable request.
