# Supplementary material for: Shockwave Therapy or Acupoint Stimulation Combined With Physiotherapy for Spasticity and Motor Function in Post‐Stroke Recovery: A Randomized Controlled Trial
Source: Brain Behav. 2026 Aug 15;16(8):e71652. doi: 10.1002/brb3.71652 (PMC13477229; doi:10.1002/brb3.71652)
Supplement: Supplementary file 1 [file BRB3-16-e71652-s001.docx]

**Supplementary file 3. Study findings visualization**

*Figure 2. Baseline characteristics visualization*

*Figure 3. Comparison of outcome measures visualization*

*Figure 4. Timeline comparison of outcome measures visualization*
