# Supplementary material for: Shockwave Therapy or Acupoint Stimulation Combined With Physiotherapy for Spasticity and Motor Function in Post‐Stroke Recovery: A Randomized Controlled Trial
Source: Brain Behav. 2026 Aug 15;16(8):e71652. doi: 10.1002/brb3.71652 (PMC13477229; doi:10.1002/brb3.71652)
Supplement: Supplementary file 2 [file BRB3-16-e71652-s002.docx]

| **Supplementary file 2. Bonferroni post-hoc pairwise comparisons of outcome measures** | | | | | | | |
| --- | --- | --- | --- | --- | --- | --- | --- |
| **Outcome** | **(I) Group** | **(J) Group** | **Mean difference (I-J)** | **Std. error** | **Sig.** | **95% Confidence Interval** | |
|  |  |  |  |  |  | **Lower bound** | **Upper bound** |
| MAS – U/L  (Baseline) | ESWT | ACU TENS | .150 | .129 | .744 | -.164 | .464 |
|  |  | UPT | .275 | .129 | .106 | -.039 | .589 |
|  | ACU TENS | ESWT | -.150 | .129 | .744 | -.464 | .164 |
|  |  | UPT | .125 | .129 | 1.000 | -.189 | .439 |
|  | UPT | ESWT | -.275 | .129 | .106 | -.589 | .039 |
|  |  | ACU TENS | -.125 | .129 | 1.000 | -.439 | .189 |
| MAS – U/L  (6 weeks) | ESWT | ACU TENS | -.175 | .138 | .620 | -.509 | .160 |
|  |  | UPT | -.450 | .138 | .004 | -.785 | -.115 |
|  | ACU TENS | ESWT | .175 | .138 | .620 | -.160 | .509 |
|  |  | UPT | -.275 | .137 | .145 | -.609 | .059 |
|  | UPT | ESWT | .450 | .138 | .004 | .115 | .785 |
|  |  | ACU TENS | .275 | .138 | .145 | -.060 | .610 |
| MAS – U/L  (18 weeks) | ESWT | ACU TENS | -.125 | .130 | 1.000 | -.439 | .189 |
|  |  | UPT | -.425 | .129 | .004 | -.739 | -.110 |
|  | ACU TENS | ESWT | .125 | .129 | 1.000 | -.189 | .439 |
|  |  | UPT | -.300 | .129 | .066 | -.614 | .014 |
|  | UPT | ESWT | .425 | .129 | .004 | .111 | .739 |
|  |  | ACU TENS | .300 | .129 | .066 | -.014 | .614 |
| MAS – L/L  (Baseline) | ESWT | ACU TENS | .125 | .137 | 1.000 | -.207 | .457 |
|  |  | UPT | .025 | .137 | 1.000 | -.307 | .357 |
|  | ACU TENS | ESWT | -.125 | .137 | 1.000 | -.457 | .207 |
|  |  | UPT | -.100 | .137 | 1.000 | -.432 | .232 |
|  | UPT | ESWT | -.025 | .137 | 1.000 | -.357 | .307 |
|  |  | ACU TENS | .100 | .137 | 1.000 | -.232 | .432 |
| MAS – L/L  (6 weeks) | ESWT | ACU TENS | -.075 | .132 | 1.000 | -.396 | .246 |
|  |  | UPT | -.350 | .132 | .028 | -.671 | -.029 |
|  | ACU TENS | ESWT | .075 | .132 | 1.000 | -.246 | .396 |
|  |  | UPT | -.275 | .132 | .119 | -.596 | .046 |
|  | UPT | ESWT | .350 | .132 | .028 | .029 | .671 |
|  |  | ACU TENS | .275 | .132 | .119 | -.046 | .596 |
| MAS – L/L  (18 weeks) | ESWT | ACU TENS | -.025 | .131 | 1.000 | -.343 | .293 |
|  |  | UPT | -.300 | .131 | .071 | -.618 | .018 |
|  | ACU TENS | ESWT | .025 | .131 | 1.000 | -.293 | .343 |
|  |  | UPT | -.275 | .131 | .114 | -.593 | .043 |
|  | UPT | ESWT | .300 | .131 | .071 | -.018 | .618 |
|  |  | ACU TENS | .275 | .131 | .114 | -.043 | .593 |
| MTS (R2–R1 angle) – U/L  (Baseline) | ESWT | ACU TENS | .349 | .508 | 1.000 | -.886 | 1.584 |
|  |  | UPT | 1.124 | .508 | .087 | -.111 | 2.359 |
|  | ACU TENS | ESWT | -.349 | .508 | 1.000 | -1.584 | .886 |
|  |  | UPT | .775 | .508 | .390 | -.460 | 2.010 |
|  | UPT | ESWT | -1.124 | .508 | .087 | -2.359 | .111 |
|  |  | ACU TENS | -.775 | .508 | .390 | -2.010 | .460 |
| MTS (R2–R1 angle) – U/L  (6 weeks) | ESWT | ACU TENS | 1.583 | .526 | .010 | .307 | 2.861 |
|  |  | UPT | 1.947 | .526 | .001 | .670 | 3.224 |
|  | ACU TENS | ESWT | -1.584 | .526 | .010 | -2.861 | -.307 |
|  |  | UPT | .363 | .526 | 1.000 | -.914 | 1.640 |
|  | UPT | ESWT | -1.947 | .526 | .001 | -3.224 | -.670 |
|  |  | ACU TENS | -.363 | .526 | 1.000 | -1.640 | .914 |
| MTS (R2–R1 angle) – U/L  (18 weeks) | ESWT | ACU TENS | 1.419 | .521 | .022 | .154 | 2.684 |
|  |  | UPT | 1.875 | .521 | .001 | .610 | 3.140 |
|  | ACU TENS | ESWT | -1.419 | .521 | .022 | -2.684 | -.154 |
|  |  | UPT | .456 | .521 | 1.000 | -.809 | 1.721 |
|  | UPT | ESWT | -1.875 | .521 | .001 | -3.140 | -.610 |
|  |  | ACU TENS | -.456 | .521 | 1.000 | -1.721 | .809 |
| MTS (R2–R1 angle) – L/L  (Baseline) | ESWT | ACU TENS | 1.026 | .555 | .201 | -.322 | 2.374 |
|  |  | UPT | 1.203 | .555 | .097 | -.145 | 2.551 |
|  | ACU TENS | ESWT | -1.026 | .555 | .201 | -2.374 | .322 |
|  |  | UPT | .177 | .555 | 1.000 | -1.171 | 1.525 |
|  | UPT | ESWT | -1.203 | .555 | .097 | -2.551 | .145 |
|  |  | ACU TENS | -.177 | .555 | 1.000 | -1.525 | 1.171 |
| MTS (R2–R1 angle) – L/L  (6 weeks) | ESWT | ACU TENS | 1.500 | .725 | .122 | -.260 | 3.260 |
|  |  | UPT | 2.625 | .725 | .001 | .865 | 4.385 |
|  | ACU TENS | ESWT | -1.500 | .725 | .122 | -3.260 | .260 |
|  |  | UPT | 1.125 | .725 | .370 | -.635 | 2.885 |
|  | UPT | ESWT | -2.625 | .725 | .001 | -4.385 | -.865 |
|  |  | ACU TENS | -1.125 | .725 | .370 | -2.885 | .635 |
| MTS (R2–R1 angle) – L/L  (18 weeks) | ESWT | ACU TENS | 1.500 | .668 | .080 | -.124 | 3.124 |
|  |  | UPT | 3.600 | .668 | .000 | 1.976 | 5.224 |
|  | ACU TENS | ESWT | -1.500 | .668 | .080 | -3.124 | .124 |
|  |  | UPT | 2.100 | .668 | .006 | .476 | 3.724 |
|  | UPT | ESWT | -3.600 | .668 | .000 | -5.224 | -1.976 |
|  |  | ACU TENS | -2.100 | .668 | .006 | -3.724 | -.476 |
| FMA – U/L  (Baseline) | ESWT | ACU TENS | -.133 | .687 | 1.000 | -1.802 | 1.536 |
|  |  | UPT | -.838 | .687 | .675 | -2.507 | .831 |
|  | ACU TENS | ESWT | .133 | .687 | 1.000 | -1.536 | 1.802 |
|  |  | UPT | -.705 | .687 | .921 | -2.374 | .964 |
|  | UPT | ESWT | .838 | .687 | .675 | -.831 | 2.507 |
|  |  | ACU TENS | .705 | .687 | .921 | -.964 | 2.374 |
| FMA – U/L  (6 weeks) | ESWT | ACU TENS | 2.461 | 1.113 | .087 | -.242 | 5.163 |
|  |  | UPT | 5.271 | 1.113 | .000 | 2.569 | 7.973 |
|  | ACU TENS | ESWT | -2.461 | 1.113 | .087 | -5.163 | .242 |
|  |  | UPT | 2.811 | 1.113 | .039 | .108 | 5.513 |
|  | UPT | ESWT | -5.271 | 1.113 | .000 | -7.973 | -2.569 |
|  |  | ACU TENS | -2.811 | 1.113 | .039 | -5.513 | -.108 |
| FMA – U/L  (18 weeks) | ESWT | ACU TENS | 3.611 | 1.207 | .010 | .680 | 6.541 |
|  |  | UPT | 7.229 | 1.207 | .000 | 4.299 | 10.160 |
|  | ACU TENS | ESWT | -3.611 | 1.207 | .010 | -6.541 | -.680 |
|  |  | UPT | 3.619 | 1.207 | .010 | .688 | 6.549 |
|  | UPT | ESWT | -7.229 | 1.207 | .000 | -10.160 | -4.299 |
|  |  | ACU TENS | -3.619 | 1.207 | .010 | -6.549 | -.688 |
| FMA – L/L  (Baseline) | ESWT | ACU TENS | .497 | .416 | .706 | -.515 | 1.508 |
|  |  | UPT | -.403 | .416 | 1.000 | -1.414 | .609 |
|  | ACU TENS | ESWT | -.497 | .416 | .706 | -1.508 | .514 |
|  |  | UPT | -.900 | .416 | .098 | -1.910 | .112 |
|  | UPT | ESWT | .403 | .416 | 1.000 | -.609 | 1.414 |
|  |  | ACU TENS | .900 | .416 | .098 | -.112 | 1.911 |
| FMA – L/L  (6 weeks) | ESWT | ACU TENS | .896 | .896 | .958 | -1.281 | 3.073 |
|  |  | UPT | 2.299 | .896 | .035 | .122 | 4.476 |
|  | ACU TENS | ESWT | -.896 | .896 | .958 | -3.073 | 1.281 |
|  |  | UPT | 1.403 | .896 | .361 | -.775 | 3.580 |
|  | UPT | ESWT | -2.299 | .896 | .035 | -4.476 | -.122 |
|  |  | ACU TENS | -1.403 | .896 | .361 | -3.580 | .775 |
| FMA – L/L  (18 weeks) | ESWT | ACU TENS | 1.200 | .919 | .582 | -1.031 | 3.431 |
|  |  | UPT | 2.734 | .919 | .011 | .502 | 4.965 |
|  | ACU TENS | ESWT | -1.200 | .919 | .582 | -3.431 | 1.031 |
|  |  | UPT | 1.534 | .919 | .293 | -.698 | 3.765 |
|  | UPT | ESWT | -2.734 | .919 | .011 | -4.965 | -.502 |
|  |  | ACU TENS | -1.534 | .919 | .293 | -3.765 | .698 |
| SS-QOL  (Baseline) | ESWT | ACU TENS | -3.245 | 2.358 | .514 | -8.972 | 2.481 |
|  |  | UPT | .568 | 2.358 | 1.000 | -5.159 | 6.294 |
|  | ACU TENS | ESWT | 3.245 | 2.358 | .514 | -2.481 | 8.972 |
|  |  | UPT | 3.813 | 2.358 | .326 | -1.914 | 9.540 |
|  | UPT | ESWT | -.568 | 2.358 | 1.000 | -6.294 | 5.159 |
|  |  | ACU TENS | -3.813 | 2.358 | .326 | -9.540 | 1.914 |
| SS-QOL  (6 weeks) | ESWT | ACU TENS | 4.270 | 3.599 | .714 | -4.472 | 13.012 |
|  |  | UPT | 12.204 | 3.599 | .003 | 3.462 | 20.947 |
|  | ACU TENS | ESWT | -4.270 | 3.599 | .714 | -13.012 | 4.472 |
|  |  | UPT | 7.934 | 3.599 | .088 | -.808 | 16.677 |
|  | UPT | ESWT | -12.204 | 3.599 | .003 | -20.947 | -3.462 |
|  |  | ACU TENS | -7.934 | 3.599 | .088 | -16.677 | .808 |
| SS-QOL  (18 weeks) | ESWT | ACU TENS | 4.605 | 2.516 | .209 | -1.506 | 10.716 |
|  |  | UPT | 12.179 | 2.516 | .000 | 6.067 | 18.290 |
|  | ACU TENS | ESWT | -4.605 | 2.516 | .209 | -10.716 | 1.506 |
|  |  | UPT | 7.574 | 2.516 | .010 | 1.462 | 13.685 |
|  | UPT | ESWT | -12.179 | 2.516 | .000 | -18.290 | -6.067 |
|  |  | ACU TENS | -7.574 | 2.516 | .010 | -13.685 | -1.462 |
| 10MWT (seconds)  (Baseline) | ESWT | ACU TENS | .946 | 1.288 | 1.000 | -2.183 | 4.075 |
|  |  | UPT | -1.215 | 1.288 | 1.000 | -4.344 | 1.915 |
|  | ACU TENS | ESWT | -.946 | 1.288 | 1.000 | -4.075 | 2.183 |
|  |  | UPT | -2.161 | 1.288 | .288 | -5.290 | .968 |
|  | UPT | ESWT | 1.215 | 1.288 | 1.000 | -1.915 | 4.344 |
|  |  | ACU TENS | 2.161 | 1.288 | .288 | -.968 | 5.290 |
| 10MWT (seconds)  (6 weeks) | ESWT | ACU TENS | -1.561 | .746 | .115 | -3.372 | .250 |
|  |  | UPT | -3.235 | .746 | .000 | -5.046 | -1.423 |
|  | ACU TENS | ESWT | 1.561 | .746 | .115 | -.250 | 3.372 |
|  |  | UPT | -1.674 | .746 | .080 | -3.485 | .138 |
|  | UPT | ESWT | 3.235 | .746 | .000 | 1.423 | 5.046 |
|  |  | ACU TENS | 1.674 | .746 | .080 | -.138 | 3.485 |
| 10MWT (seconds)  (18 weeks) | ESWT | ACU TENS | -1.815 | .656 | .020 | -3.409 | -.220 |
|  |  | UPT | -3.717 | .656 | .000 | -5.311 | -2.122 |
|  | ACU TENS | ESWT | 1.815 | .656 | .020 | .220 | 3.409 |
|  |  | UPT | -1.902 | .656 | .013 | -3.496 | -.3080 |
|  | UPT | ESWT | 3.717 | .656 | .000 | 2.122 | 5.311 |
|  |  | ACU TENS | 1.902 | .656 | .013 | .308 | 3.496 |
| One-way ANOVA test.  Acu-TENS: Acupoint-based Transcutaneous Electrical Nerve Stimulation; ESWT: Extracorporeal Shockwave Therapy; FMA: Fugl-Meyer Assessment; MAS: Modified Ashworth Scale; MTS: Modified Tardieu Scale; SS-QOL: Stroke-Specific Quality of Life; UPT: Usual Physiotherapy Treatment; 10MWT: 10-Meter Walk Test. | | | | | | | |
