# Supplementary material for: Shockwave Therapy or Acupoint Stimulation Combined With Physiotherapy for Spasticity and Motor Function in Post‐Stroke Recovery: A Randomized Controlled Trial
Source: Brain Behav. 2026 Aug 15;16(8):e71652. doi: 10.1002/brb3.71652 (PMC13477229; doi:10.1002/brb3.71652)
Supplement: Supplementary file 3 [file BRB3-16-e71652-s003.docx]

| Supplementary file 1. Mixed within-group analysis | | | | | | | | | |
| --- | --- | --- | --- | --- | --- | --- | --- | --- | --- |
| Outcome measures | **6 weeks – Baseline** | | | **18 weeks – Baseline** | | | **18 weeks – 6 weeks** | | |
|  | ESWT group  (n = 40) | Acu-TENS group  (n = 40) | UPT group  (n = 40) | ESWT group  (n = 40) | Acu-TENS group  (n = 40) | UPT group  (n = 40) | ESWT group  (n = 40) | Acu-TENS group  (n = 40) | UPT group  (n = 40) |
| MAS – U/L | -1.35 (0.80) | -1.03 (0.73) | -0.63 (0.61) | -1.13 (0.79) | -0.85 (0.70) | -0.43 (0.64) | 0.23 (0.48) | 0.18 (0.68) | 0.20 (0.41) |
| MAS – L/L | -1.05 (0.81) | -0.85 (0.77) | -0.68 (0.70) | -0.88 (0.69) | -0.73 (0.68) | -0.55 (0.68) | 0.18 (0.38) | 0.13 (0.33) | 0.12 (0.46) |
| MTS (R2 - R1 angle) – U/L | 3.55 (2.38) | 2.31 (2.77) | 2.73 (2.50) | 2.78 (2.57) | 1.71 (2.86) | 2.03 (2.56) | -0.77 (0.95) | -0.61 (1.32) | -0.70 (1.26) |
| MTS (R2 - R1 angle) – L/L | 4.42 (3.60) | 3.95 (3.98) | 3.00 (3.75) | 3.67 (3.51) | 3.20 (3.87) | 1.28 (4.07) | -0.75 (1.58) | -0.75 (1.68) | -1.73 (2.25) |
| FMA – U/L | 13.42 (6.99) | 10.83 (6.07) | 7.31 (5.48) | 17.22 (6.20) | 13.48 (6.80) | 9.15 (5.96) | 3.80 (4.90) | 2.65 (3.68) | 1.84 (4.81) |
| FMA – L/L | 8.21 (3.72) | 7.81 (4.92) | 5.51 (4.79) | 10.53 (3.89) | 9.83 (5.16) | 7.39 (4.60) | 2.32 (4.13) | 2.02 (2.90) | 1.89 (2.96) |
| SS-QOL | 23.10 (17.41) | 15.59 (17.37) | 11.47 (21.10) | 26.67 (15.22) | 18.82 (15.36) | 15.06 (15.35) | 3.57 (6.87) | 3.23 (7.14) | 3.59 (10.58) |
| 10MWT (seconds) | -5.97 (6.21) | -3.95 (7.73) | -3.47 (5.59) | -6.90 (5.40) | -4.40 (7.75) | -4.14 (5.46) | -0.93 (2.11) | -0.44 (1.16) | -0.67 (1.24) |
| Acu-TENS: Acupoint-based Transcutaneous Electrical Nerve Stimulation; ESWT: Extracorporeal Shockwave Therapy; FMA: Fugl-Meyer Assessment; MAS: Modified Ashworth Scale; MTS: Modified Tardieu Scale; SS-QOL: Stroke-Specific Quality of Life; UPT: Usual Physiotherapy Treatment; 10MWT: 10-Meter Walk Test. | | | | | | | | | |
